# Genome-wide occupancy reveals the localization of H1T2 (H1fnt) to repeat regions and a subset of transcriptionally active chromatin domains in rat spermatids

**DOI:** 10.1186/s13072-020-00376-2

**Published:** 2021-01-06

**Authors:** Vasantha Shalini, Utsa Bhaduri, Anjhana C. Ravikkumar, Anusha Rengarajan, Rao M. R. Satyanarayana

**Affiliations:** 1 From the Chromatin Biology Laboratory, Molecular Biology and Genetics Unit, Jawaharlal Nehru Centre for Advanced Scientific Research, Jakkur P.O., Bangalore, 560064 India; 2 Department of Life Sciences, University of Trieste, Trieste, Italy; 3 European Union’s H2020 TRIM-NET ITN, Marie Sklodowska-Curie Actions (MSCA), Leiden, The Netherlands

**Keywords:** Spermiogenesis, Linker histone, Spermatid, ChIP-sequencing, Histone PTMs

## Abstract

**Background:**

H1T2/H1FNT is a germ cell-specific linker histone variant expressed during spermiogenesis specifically in round and elongating spermatids. Infertile phenotype of homozygous H1T2 mutant male mice revealed the essential function of H1T2 for the DNA condensation and histone-to-protamine replacement in spermiogenesis. However, the mechanism by which H1T2 imparts the inherent polarity within spermatid nucleus including the additional protein partners and the genomic domains occupied by this linker histone are unknown.

**Results:**

Sequence analysis revealed the presence of Walker motif, SR domains and putative coiled-coil domains in the C-terminal domain of rat H1T2 protein. Genome-wide occupancy analysis using highly specific antibody against the CTD of H1T2 demonstrated the binding of H1T2 to the LINE L1 repeat elements and to a significant percentage of the genic regions (promoter-TSS, exons and introns) of the rat spermatid genome. Immunoprecipitation followed by mass spectrometry analysis revealed the open chromatin architecture of H1T2 occupied chromatin encompassing the H4 acetylation and other histone PTMs characteristic of transcriptionally active chromatin. In addition, the present study has identified the interacting protein partners of H1T2-associated chromatin mainly as nucleo-skeleton components, RNA-binding proteins and chaperones.

**Conclusions:**

Linker histone H1T2 possesses unique domain architecture which can account for the specific functions associated with chromatin remodeling events facilitating the initiation of histone to transition proteins/protamine transition in the polar apical spermatid genome. Our results directly establish the unique function of H1T2 in nuclear shaping associated with spermiogenesis by mediating the interaction between chromatin and nucleo-skeleton, positioning the epigenetically specialized chromatin domains involved in transcription coupled histone replacement initiation towards the apical pole of round/elongating spermatids.

## Background

The production of mammalian spermatozoa is a precisely controlled, complex and dynamic developmental process involving the differentiation of diploid spermatogonia to spermatocytes and finally to haploid round spermatids. The last phase of spermatogenesis namely, spermiogenesis comprises a series of morphological transformations and extreme condensation of the chromatin to form highly condensed, polarized spermatozoa. Spermiogenesis is divided into different steps based on the three developmental processes involving condensation of the nucleus followed by the formation of the flagellum and the acrosome. During the steps 1–7, the early round spermatids are characterized by a round nucleus, with the beginning of acrosome and axoneme assembly. These early round spermatids actively transcribe many of the mRNAs necessary for the whole process of spermiogenesis, while many of these mRNAs are subjected to delayed translation until the later stages of spermiogenesis and sperm function. The step 8 comprises the acrosome polarization to one side of the nucleus and the initiation of spermatid elongation followed by the assembly of the accessory structures needed for flagella function. The sperm transcripts remain as stable as ribonucleoprotein particles (RNPs) contributing to the final sperm proteome, which can have more relevant functional roles in the zygote or early embryo [1, 2]. The final stage of spermiogenesis is the spermiation, a process in which elongating spermatids undergo extensive chromatin remodeling, which have been well studied in mouse [3].

Chromatin remodeling is a unique process occurring during spermiogenesis, wherein 90% of core histones are being replaced by the testes-specific histone variants, then transition proteins (TPs), and eventually protamines (PRMs) [4, 5]. The process is initiated by the incorporation of testes-specific histone variants and somatic histone variants, and concurrent histone post-translational modifications [6, 7]. Together with histone PTMs like acetylation, ubiquitination, methylation and phosphorylation, many variants of histone H1 (H1t, H1T2 HILS1), H2A and H2B (TH2A, TH2B, ssH2B, H2A.B.bd, H2BL1, H2BL2, H2AL1-H2AL3) and H3 (H3t, H3.3) play a major role in the extensive chromatin remodeling events leading to the generation of mature sperm [8, 9]. Histone replacement starts with the hyperacetylation of histone H4 (lysine residues at K5, 8, 12 and 16) at step 8 just before the elongation of spermatids, and lack of H4 hyperacetylation leads to impaired spermatogenesis in mouse [10, 11]. Transition proteins incorporation is observed in steps 10 and 11, followed by protamine 1 at step 11 and protamine 2 at step 12 [12]. In addition to the nuclear chromatin condensation, sperm head shaping is a tightly regulated process in spermiation, which involves accurate assembly of cytoskeletal (manchette) and cytoplasmic (acrosome) structures as demonstrated by many mouse genetic studies [13, 14]. Until recently it was believed that transcriptionally inert genetic material is transferred to the embryo. However, more recent studies have suggested mature sperm as carriers of epigenetic information to the next generation by retaining typical covalent histone modifications at important genomic loci and a three-dimensional architecture similar to that of somatic cells [15, 16].

Many histone variants are expressed during spermiogenesis and modulate the chromatin structure to facilitate the histone-to-protamine replacement process [17, 18]. Linker histones primarily function in the formation and stabilization of the higher-order chromatin structure [19]. The H1 family proteins are greatly divergent in their structure, especially in the C terminal domain which influences the chromatin binding efficiency of these linker histones [20]. The C-terminal domain of germ cell-specific linker histones is more divergent with the formation of additional alpha helices in comparison to other H1s [21]. Spermiogenesis begins with the extensive restructuring of sperm chromatin during the meiotic prophase interval when the somatic H1s are replaced by the variant H1t. H1t is subsequently replaced by another distinct linker histone H1T2 in round spermatids which is specifically localized at the apical pole in the nucleus of round and elongating spermatids (steps 5–15 of mouse spermiogenesis) which disappears during the maturation of testicular spermatozoa. H1T2 has been identified as a critical histone variant necessary for spermiogenesis as the mutant males are infertile due to delayed nuclear condensation and aberrant elongation of spermatids [22, 23]. HILS1 is another linker histone variant which is specifically expressed in elongating and elongated spermatids (steps 9–15 of mouse spermiogenesis). Earlier studies on linker histone variants were mainly restricted to biochemical and biophysical investigations which showed that they generate subtle different higher order structures in vitro. More recently, focused studies have been initiated to understand how the sequence diversity of H1 proteins leads to distinct chromatin structures associated with different functional properties. Studies from our laboratory recently focused on the mechanism by which testes-specific H1 variants contribute to diverse chromatin function by studying their genomic localization, PTMs and chromatin loci specific interacting proteins [24, 25]. Linker histone H1t constituting about 50–60% of total H1 content induces local chromatin relaxation to recruit the appropriate protein machinery for establishing closed chromatin repressed structures [25]. An earlier study from our laboratory had shown that HILS1, whose expression is mainly confined to the elongating spermatids is involved in the maintenance of repressed chromatin architecture by preferential binding to LINE element containing chromatin domains in rat spermatids. Unlike H1t and HILS1 whose absence can be possibly compensated by other histone variants, H1FNT (H1T2) that appears in between H1t and HILS1 has been shown to be an essential linker histone for the packaging of sperm chromatin and for the formation of the compact sperm nucleus that is required for fertilization as revealed from the infertile phenotype in homozygous H1T2/H1FNT mutant males [23]. In the present study, we have addressed the potential genomic function(s) of H1T2 by analyzing the genome wide occupancy of histone H1T2 and loci specific histone PTM signature and associated chromatin proteins.

## Results

### H1FNT/H1T2—a highly divergent germ cell-specific linker histone

**Fig. 1:**
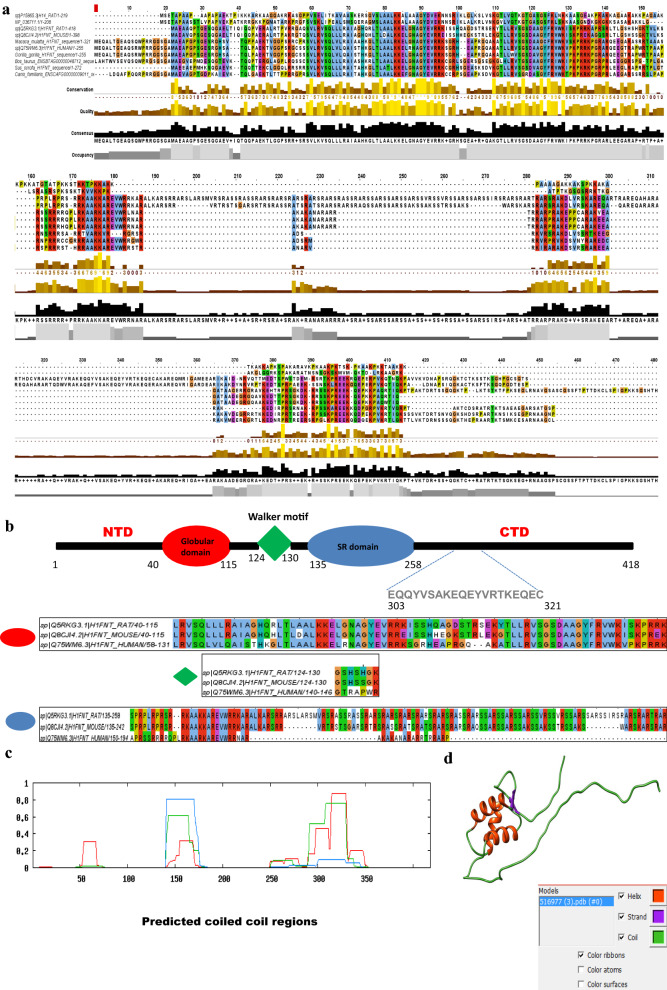
H1T2 possess highly divergent C terminal domain. **a** Multiple sequence alignment of sequences of linker histone variants. Sequences from either somatic linker histone H1d or germ cell-specific H1t were aligned to consensus sequence from germ cell-specific H1T2 of different species. Amino acids are colored according to their similarity degree using the ClustalX option of Jalview. **b** Schematic representation of domain architecture of rat H1T2 protein: Globular domain (red), ATP-binding walker motif (green), serine-arginine rich domain (blue). Peptide sequence against which the antibody is raised, is highlighted in the C terminal domain. Clustal alignment of H1T2 sequence from rat, mouse and human species is also shown in the figure. Conserved globular domain, walker motif (ATP binding), and the divergent SR domain in the H1T2 sequences are represented separately. **c** Coiled-coil prediction analysis of rat H1T2 sequence (https://embnet.vital-it.ch/cgi-bin/COILS_form_parser). **d** Secondary structure of the rat H1T2 sequence predicted using Phyre2 (PDB format)

Similar to all metazoan linker histones, H1T2 also possess a short, flexible N-terminal tail domain (NTD), a central globular domain (GD) with a winged-helix motif (three α helix) and a highly extended C-terminal tail domain (CTD). Figure 1a summarizes the comparison of H1T2 sequences from different mammalian species. A comparison of sequences of H1T2 with H1.4 (somatic) or H1t (germ-cell specific) reveals that H1T2 is more diverged (≤ 21% similarity) from other linker histones. The human orthologue of H1T2 has highly diverged from rodents, with only 37.6% homology between mouse and human H1T2 sequence, 38.4% similarity between rat and human H1T2, while sharing most similarity with the gorilla orthologue with 97.6% identity. The highly extended C-terminal domain of H1T2 consists of a Walker motif (ATP/GTP binding), SR domain with SR repeats and many phosphorylation sites (Fig. 1b). At the same time, H1T2 proteins lack the 16-mer (S/TPKK) motif, which is known to have DNA condensation properties [26]. In the globular domain, human H1T2 shares aminoacid identity at several positions with the rodent specific H1T2, whereas the ATP-binding motif and extended C-terminus including the SR domain are not present (Fig. 1b). It is interesting to note that while the general property of the C-terminal domain of linker histone is to stabilize higher order chromatin folding, functional sub-domains which are present in the CTD of rodent specific H1T2 are absent in higher mammals. Comparative study of testis protein evolution in rodents identified H1T2 as one of the rapidly evolving testis-expressed proteins [27]. H1T2 possesses a putative winged-helix domain consisting three alpha helices in addition to a unique coiled-coil domain in the C-terminal domain (Fig. 1c, d). A similar domain is reported in the N-terminal domain of Drosophila linker histone Mst77f, which is responsible for the DNA aggregation property of the H1 [28]. Direct alignment of Mst77f with either HILS1 or H1T2 failed to uncover any significant sequence conservation with less than 15% identity, even though HILS1 has been referred in the literature as a putative mammalian homologue of Mst77f [28]. Extensive studies on *Drosophila* MSt77f revealed dual roles in spermatogenesis, firstly involved in regional distribution of chromatin beneath the stripe of microtubules in histone-based nuclei, and secondly, it is essential for nuclear shaping through the stabilization of microtubules [9, 28]. We found a high degree of similarity between Mst77f and H1T2 proteins with respect to coiled-coil domains. Numerous studies have identified H1T2 as a critical testes-specific histone H1 for spermiogenesis, lack of which results in reduced fertility because of delayed nuclear condensation and aberrant elongation characterized by acrosome detachment and fragmented DNA [22]. With this background, we were curious to gain more insights into the genomic functions of H1T2 in the context of our recent studies on the other two testes-specific histone variants, H1t and HILS1 [24, 25].

### Characterization of antibody against CTD of rat H1T2 protein

**Fig. 2:**
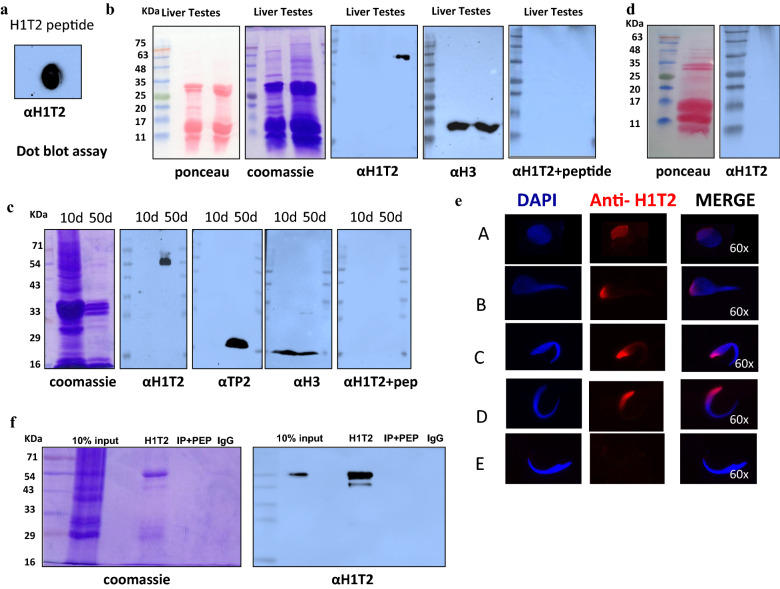
Characterization of antibody raised against the highly divergent C terminal domain of H1T2. **a** Dot-blot analysis of affinity purified antibody against H1T2 with respective peptide. **b** Western blot analysis of the acid extracts from rat liver (*lane 1*) and testes (*lane 2*) with antibody against H1T2. **c** Western blot analysis of acid-soluble proteins from 10 days postnatal (*lane 1*) and 40 days postnatal (*lane 2*) rat testes nuclei with anti-H1T2 antibodies in the presence or absence of peptide competition with a 200-fold molar excess of the peptide used for antibody generation, as indicated. Western blot analysis with anti-TP2 and anti-H3 antibodies served as positive controls. **d** Western blot analysis of acid extracts of epididymal sperm using H1T2 antibody. **e** Immunofluorescence analysis in spermatids using H1T12 antibody. **f** Western blot analysis of the pull-down fractions with α H1T2. Input (lane 1), anti-H1T2 antibody (lane 2), anti-H1T2 antibodies + peptide (lane 3), IgG (lane 4). No signal was observed in peptide competition and pre-immune IgG lanes

Since H1T2 has been shown to occupy specialized chromatin regions in the apical region of spermatids, we wanted to analyze the genomic domains bound to H1T2 within these regions by ChIP-sequencing analysis. Because of the unavailability of a ChIP grade commercial antibody against the linker histone H1T2, we raised in house antibody against the C-terminal domain of rat H1T2, using the synthetic peptide (303-EQQYVSAKEQEYVRTKEQEC-321) (Fig. 1b), which has shown earlier to be a strong immunogen [22]. As expected, a strong immunoreactive signal was observed with dot-blot analysis using the peptide as an antigen when probed with the affinity purified antibody (Fig. 2a). Western blotting analysis was performed with the tissue lysate from both liver and testes of 35- to 50-day-old rats. The antibodies reacted strongly with the testicular lysate with a distinct band at 54 kDa. In contrast, the antibody failed to recognize any of the somatic H1 subtypes in the liver lysate, confirming the specificity of the antibody (Fig. 2b). There was no signal observed in the peptide competition control lane, wherein the antibody was pre-incubated with 200-fold molar excess of the immunogen. H3 antibody was used as a positive control in these experiments, wherein we observed a positive signal for both liver and testes lysate. Western blot analysis was also performed with 25 and 50 day old rat testes acid extracts, wherein a strong H1T2 signal was observed with the 50 day old rat testes extract (contributed predominantly by round/elongating spermatids). 10-day-old rat testes lysate acted as a negative control for the experiment, which were contributed predominantly by somatic cells and spermatogonia (Fig. 2c). Taken together, these experiments confirm that the antibody raised against the C terminal domain of rat H1T2 reacts specifically with the germ cell-specific linker histone H1T2, and does not react with any other linker histone variants. Additionally, H1T2 signals were not detected in the histone extracts of mature sperm collected from rat epididymis (Fig. 2d). Further immunolocalization studies of H1T2 in round and elongating spermatids revealed typical localization pattern with a cap like structure in the polar region of round spermatids, agreeing with the already established localization pattern of H1T2 signal at the nuclear region beneath the acrosome (Fig. 2e) [29].

### H1T2 binds to distinct genomic domains in round/elongating spermatids

**Fig. 3:**
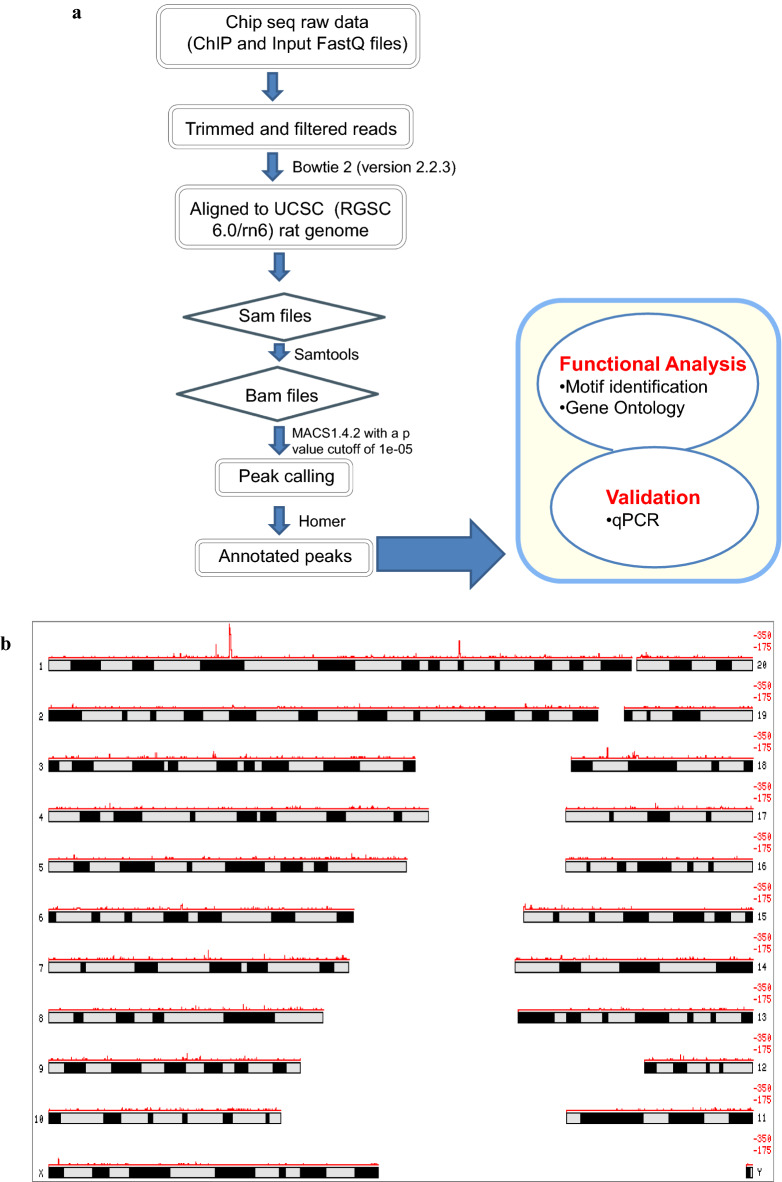

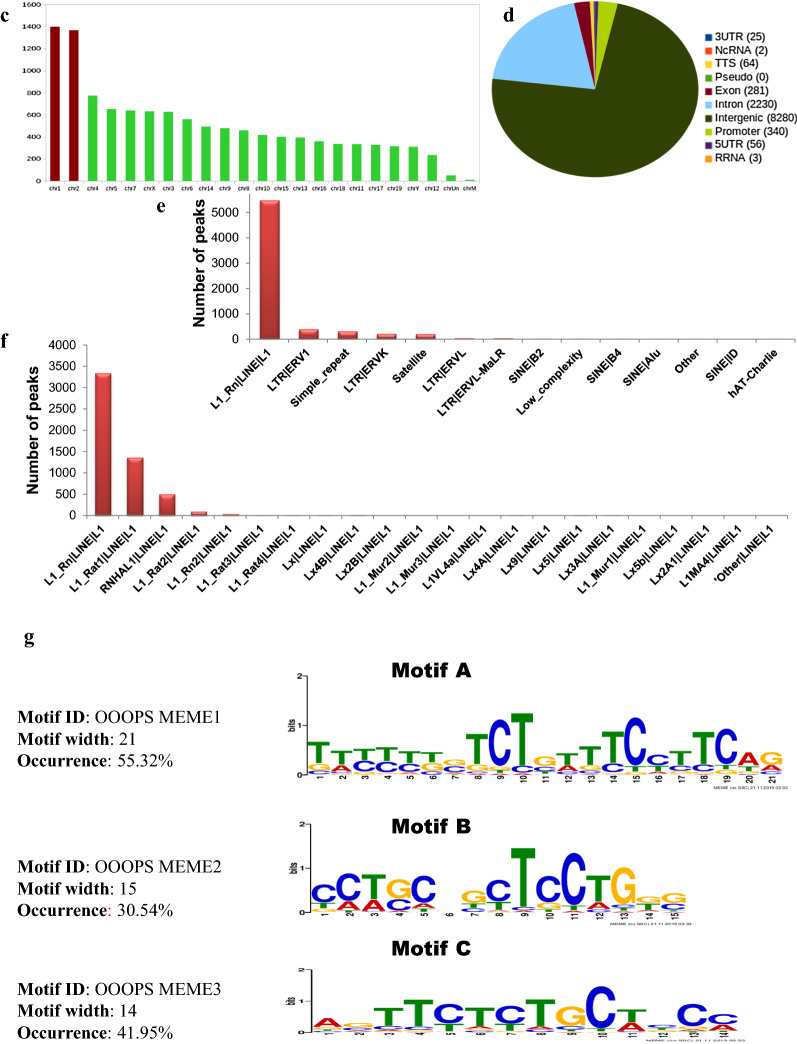
Genome-wide analysis of H1T2 in rat spermatid genome using antibody against C terminal domain. **a** Schematic representation of the protocol used for ChIP-sequencing analysis. **b** Chromosome-wise enrichment of H1T2 ChIP peaks in rat spermatid genome. **c** Number of peaks distributed across different rat chromosomes (x-axis), where y-axis represents the number of peaks. Box plot representing the H1T2-associated average peak length (y-axis) across different chromosomes (x-axis). **d** Pie chart distribution of H1T2 peaks for various genomic features in rat spermatids. The peaks are distributed in among intergenic, intron, promoter, exon, TTS, 5′UTR, 3′UTR regions. *I,* bar diagram of number of occurrences of peaks across repeat elements. **e** Bar diagram showing the number of occurrences of peaks across different classes of repeat elements. **f** Bar diagram showing the number of occurrences of peaks across different subclasses of LINE-1 repeat elements. **g** Motif identification by MEME. The figure represents the details of the three most significant motifs identified from the overlapping peak summits (H1T2 ChIP peaks)

ChIP-sequencing was performed using the highly specific H1T2 antibody in rat round/elongating spermatids chromatin. Chromatin samples were collected from 35- to 50-day-old rat testes (with a major population being represented by round and elongating spermatids). Cross-linked chromatin was subjected to sonication for 45 cycles (15 s on/15 s off cycle), followed by immunoprecipitation using H1T2 antibody. Western blot analysis confirmed the immunoprecipitation with a positive signal at 54 kDa both in the input and IP lanes, whereas the antibody was unable to recognize any protein in the peptide competition lane (antibody was competed with ~ 200 fold molar excess of the immunogen). Non-specific IgG also acted as a negative control for the H1T2 immunoprecipitation experiment (Fig. 2f). H1T2 immunoprecipitated DNA from the soluble extract of rat round/elongating spermatids was subjected to next-generation sequencing using Illumina HiSeq X Ten system using paired-end reads of 150-nt length. After preprocessing the raw reads, the clean reads were aligned against UCSC *Rattus norvegicus* genome (rn6) using Bowtie2 (version 2.2.3) (Fig. 3a). To analyze the extent of H1T2 occupancy in the spermatid genome, genomic regions associated to H1T2 was determined by MACS1.4.2 software with a p value cutoff of 1e−05. A total of 11,570 enriched regions were obtained for H1T2 and the chromosome-wise plots were generated (Fig. 3b; Additional file 1: Table S1). The number of regions bound to H1T2 is maximally associated with chromosome 1 and 2, whereas the remaining peaks were evenly distributed across other chromosomes (Fig. 3c; Additional file 2: Table S2). We have also analyzed the peak length distribution and fold enrichment across different chromosomes and the data is represented in the supplementary section (Additional file 3: Figure S1, Additional file 4: Table S3, Additional file 5: Figure S2, Additional file 6: Table S4) To analyze the distribution of H1T2 peaks in various genomic domains of rat spermatid genome, H1T2 enriched genomic regions were annotated and categorized into CpG islands, repetitive elements, exons, introns, intergenic regions, 3′UTR and 5′UTR using HOMER v4.7 (Fig. 3d). H1T2 enriched regions showed a bias towards the intergenic regions (71.56%), while at the same time a significant number of peaks were in the genic regions [promoter (2.94%), 5′ UTR (0.47%), 3′ UTR (0.22%), exon (2.43%), intron (19.27%)]. Next the repeat density in the H1T2-associated intergenic regions was calculated separately for interspersed repeat types including LINEs and LTRs (long autonomous retrotransposons) and SINEs (nonautonomous short < 300 bp retrotransposons that co-opt the LINE transposition machinery) [30]. The peak density is much lower in SINE/LTR regions compared to LINE rich region, with around 66% of H1T2 bound intergenic regions being LINE repeats (Fig. 3e; Additional file 7: Table S5). Traditional views considered spermatozoa as transcriptionally silent cells, especially LINE repeats as the successful retrotransposons which are epigenetically repressed by CpG DNA methylation in conjunction with the piRNA pathway with multiple epigenetic mechanisms [31]. Earlier studies from our laboratory had analyzed the epigenetic status of the LINE repeats at different stages of spermatogenesis in the context of linker histones occupancy. In pachytene spermatocytes, H1t decorates the methylated LINE and LTR repeats in addition to heterochromatin repressive marks [25]. As the sperm differentiation progresses through histone hyper acetylation and incorporation of other histone variants, HILS1 gets incorporated to provide a more loosened chromatin to facilitate the histone replacement, still maintaining the repressed chromatin architecture of the majorly bound LINE repeats [24]. An interesting observation from this study is the association of H1T2 to a subset of promoter-TSS chromatin domains in spermatids (~ 3% of total CHIP-seq reads). In contrast, only 0.6 and 0.08% of the ChIP-seq reads were associated with promoter regions by H1t and HILS1, respectively [24, 25]. Detailed annotation of the H1T2 bound LINE repeats revealed the major association to a particular class of LINE repeat, LINE L1 Rn (Fig. 3f; Additional file 8: Table S6) similar to our previous observation with respect to HILS1 [24]. Full-length rat L1s (L1Rn) with intact open reading frames (ORFs) is considered as the functional master copies for retrotransposition. In addition, a recent study has reported strong L1Rn RNA expression and hypomethylation status in rat testes [32]. By using the motif analysis program MEME-ChIP, we identified three consensus motifs to be significant in the total H1T2 peaks (Fig. 3g; Additional file 9: Table S7) and the percentage of occurrence of each of the motif were found to be 55.32% (Motif A), 30.54% (Motif B) and 41.95% (Motif C).

### Functional annotation of the H1T2-associated genomic domain of spermatid genome

**Fig. 4:**
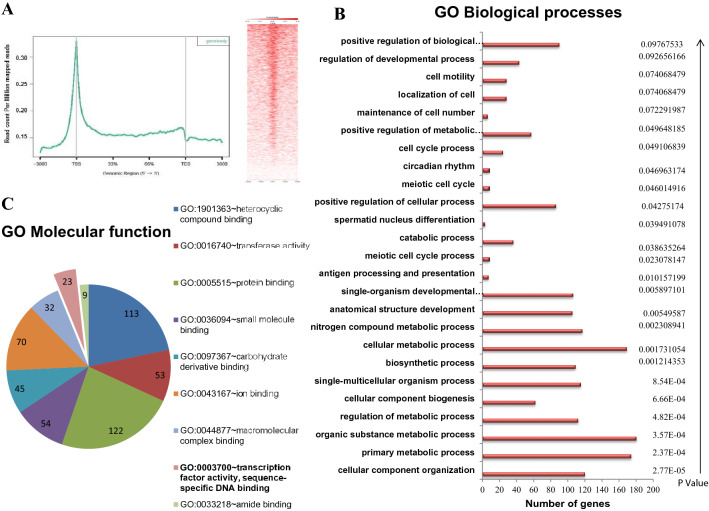

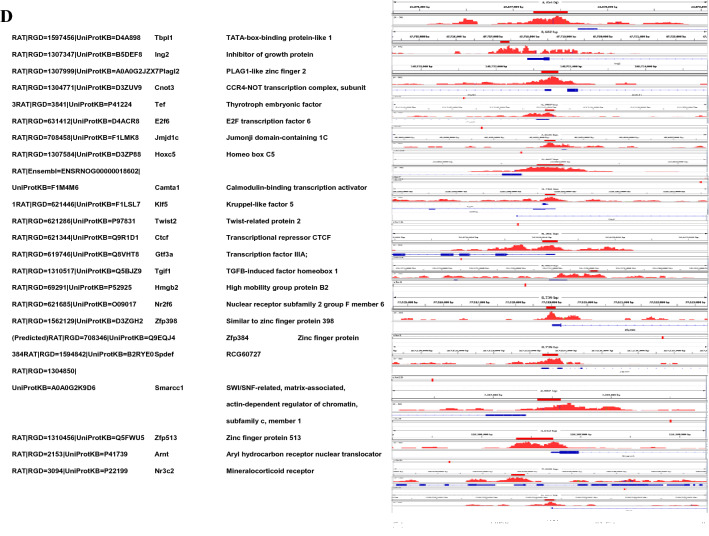
Functional analysis of H1T2 bound promoter-TSS regions in rat spermatids genome. **a** Gene ontology analysis of the 321 genes whose promoter-TSS regions are bound to H1T2. David software was used for the analysis and the results are shown in the figure. GO biological processes are represented in the figure with P values. X-axis represents the number of genes associated to the GO term. **b** Distribution of H1T2 peaks from ± 3 kb centered around TSS of genes in the spermatids genome and heatmap showing the same observation ngs. Plot showing distribution of H1T2 peaks throughout the gene. **c** GO analysis of 321 genes according to molecular function represented as pie diagram. The number of genes associated to the GO term is given in the pie chart. 23 transcription factors whose promoter-TSS is bound to H1T2 are highlighted in the pie diagram. **d** Integrative Genomics Viewer (IGV) outputs of representative genomic regions showing the occupancy of H1T2 at various transcription factors of the spermatid genome

It was most surprising that, ChIP-sequencing analysis identified 321 gene promoters as the potential binding sites for H1T2 predominantly between ( ±) 3 Kb from transcription start site in rat round/elongating spermatids. When we carried out distribution analysis of our ChIP peaks within the ± 3 kb region centered around TSS of genes, the distribution pattern turned into a sharp bell-shaped curve, showing a peak at the TSS position (Fig. 4a). To gain insights into these regions, we carried out gene ontology analysis of the genes associated with these promoters (Fig. 4b; Additional file 10: Table S8). Maximum number of genes were classified to be involved in metabolic process associated with spermatogenesis (180 genes), which have been reported to be associated with the active mark H3K4me3 in both human and mouse spermatid genome [33, 34]. The most significant category was found to be belonging to cellular component organization with a p value of 2.7E−0.5. Interestingly, among these we found three important genes namely *HMGB2, PYGO1* and *TBPL1* which are known to be required for the spermatid nuclear differentiation. *HMGB2* and *TBPL1* are already identified as essential chromatin organizers that are necessary for proper chromocenter formation and/or maintenance, as revealed from targeted mutagenesis studies [35]. In another study, loss of HMGB2 in mice affected H1T2 localization in late round spermatids and revealed intranuclear chromatin organization is critical for correct polar localization of H1T2 [29]. Additionally, we also found four other genes *ASH1*, *CECR2*, *BRD2* and *BRD4* belonging to bromodomain class of proteins, which are considered as the master regulators of maintenance of intact chromocenter in spermatids (Additional file 1: Table S1) [35].

Among the promoter-centric H1T2-associated genes, we observed that 23 genes belonged to transcription factor family upon GO: molecular function analysis (Fig. 4c; Additional file 11: Table S9). The identified occupancy peaks for these 23 transcription factors are shown in Fig. 4d. As mentioned above, TATA box‐binding protein‐like 1 (TBPL1) is one among these, which has already been identified to be important for male sterility as evidenced by the sterile phenotype in *Tbpl1*^−/−^ male mice due to a late and complete arrest of spermiogenesis at step 7 in stage VII seminiferous tubules [36]. *ING2*, another candidate transcription factor whose promoter is bound to H1T2 is reported to be an essential regulator of mammalian spermatogenesis which is characterized by an increased expression level in testes and infertile knockout male mice. Furthermore, a decreased *ING2* expression level is also associated with defective spermatogenesis and male infertility in humans [37]. *E2F6*, a part of the mammalian polycomb complex, is another transcription factor, expressed in the later stages of spermatogenesis, which has been identified as a master regulator through the coordinated action as both repressor and activator depending on the stage of expression. During male germ cell meiosis, *E2F6* acts as an activator of meiosis-specific genes, demonstrated from moderately impaired spermatogenesis in *E2F6* knockout mice [38]. E2F6 recruits polycomb group proteins to function as a repressor of target genes during embryo development specifically associated to developmental patterning [39, 40]. It is also essential for the long-term somatic silencing of certain male germ cell-specific genes and dispensable for cell cycle regulation [41]. H1T2 associates with the promoter of *JMJD1C* gene*,* which plays an indispensable role in mouse spermatogenesis as Jmjd1c-deficient males became infertile [42]. It contributes to the long-term maintenance of the male germ line by promoting spermatogonial stem cell self-renewal by up-regulating *Oct4* expression [43], required for mouse embryonic stem cell (ESC) self-renewal, mechanistically with the help of pluripotency factor KLF4 and maintains ESC identity and somatic cell reprogramming. Surprisingly, the promoter of KLF4, a pluripotency transcription factor which is highly expressed in male post-meiotic germ cells is also bound to H1T2, which is however, dispensable for spermatogenesis. It is interesting that lack of KLF4 alone in male germ cells does not prevent spermiogenesis and male fertility [44]. Deletion of *Klf4* in male mouse germ cells has been shown to affect the post-meiotic transcriptome including defined transcriptional regulators and identified as a key factor necessary for postnatal development and differentiation of the mouse testes [45]. *CAMTA1*, one among the CAMTA gene family of transcription factors whose promoter is occupied by H1T2, is an important gene in both spermatogenesis and embryo development. CAMTA1 gene is important for various processes of fertility namely motility, capacitation, and acrosome reaction through calmodulin mediated calcium regulation. Additionally it is important for embryonic heart development, which is crucial for proper embryogenesis [46]. The TSS region of *CTCF*, an architectural protein governing chromatin organization in sperm genome is also associated with linker histone H1T2, whose inactivation in male germ cells (*Ctcf*-cKO mice) resulted in impaired spermiogenesis and infertility [47]. Interestingly, this study has also shown that H1T2 is one among those genes which is down-regulated in *Ctcf*-cKO mice and displays the same phenotypic features of H1T2 deleted condition namely abnormal head morphology, aberrant chromatin compaction, impaired protamine 1 incorporation [47]. Being a highly conserved Zn finger protein, CTCF is critical for the three-dimensional organization of the genome and related functions during the development of various cell and tissue types, ranging from embryonic stem cells and gametes, to neural, muscle and cardiac cells [48].

Recent evidences accumulating in the literature are suggesting that sperm is programmed to support proper embryonic expression of genes encoding important embryonic regulators and in this context spermatids contribute epigenetic information required for proper embryonic gene expression [49]. Our GO enrichment analysis also showed enrichment of H1T2 ChIP signals at the promoters of genes of several other developmental processes related pathways (Fig. 4a; Additional file 10: Table S8): GO: 0044707 ~ single-multicellular organism process (115 genes); GO: 0048856 ~ anatomical structure development (105 genes); GO: 0044767 ~ single-organism developmental process (106 genes); GO: 0050793 ~ regulation of developmental process (43 genes). Some of the important candidate genes are *TWIST2, TGLF1, CNOT3, SMARCC3* and *ZFP* whose functions in early embryonic differentiation process have been well documented [50–54].

### Validation of H1T2 ChIP peaks of novel target genes by ChIP-PCR

**Fig. 5:**
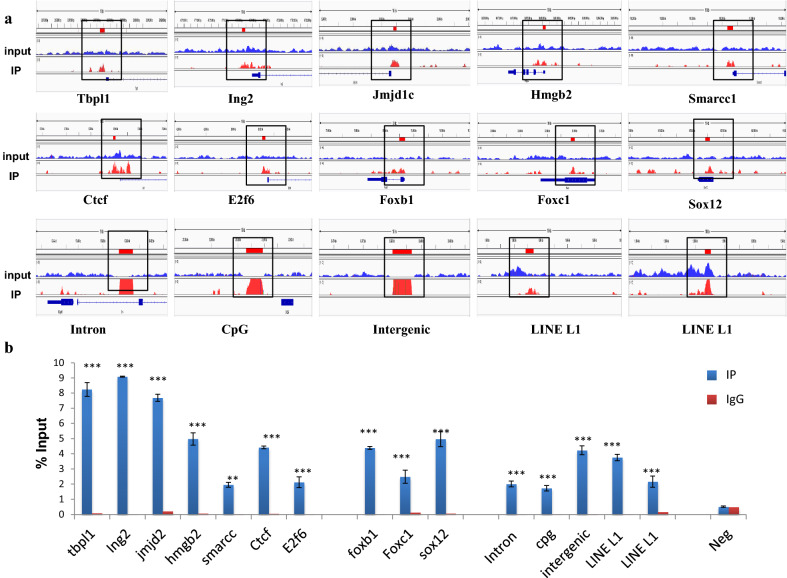
Validation of H1T2 ChIP-sequencing data by ChIP PCR analysis of representative genes bound to the linker histone H1T2. **a** Screenshots of H1T2 ChIP peaks displayed from Integrative Genomics Viewer. Shown are 15 different genomic regions associated to H1T2. The y-axis unit is reads per million (rpm).The x-axis represent the genomic positions in base pairs as follows: Chr1:23977211–23977579; Chr16:47717217–47717457; Chr20:22882800–22883032; Chr16:36080014–36080219; Chr8:118206347–118206725; Chr19:37599804–37600003; Chr6:42092121–42092366; Chr8:75994988–75995401; chr17:33949714–33950006; Chr3:147864851–147865149; Chr5:173429327–173430483; Chr1:213008609–213009881; Chr15:33478042–33479362; Chr5:1682868–1683456; ChrY:1588659–1589079. **b** Bar graphs showing H1T2 ChIP-PCR results represented as % of input. The first seven regions represents the H1T2 occupancy in transcription factors and the next group of three regions represent the H1T2 bound developmental genes while the remaining regions shows the preferential binding of H1T2 to the repeat elements. A negative control region (specific regions of chr 15) was also included for ChIP PCR, where no peaks were present according to the data analysis and visualization. Results are from three independent experiments and error bars represent standard deviation. *shows comparison with IgG control and # shows comparison with IP + pep group. ***(P ≤ 0.001), **(P ≤ 0.01), *(P ≤ 0.05)

We selected a representative set of 15 ChIP peaks for further validation of H1T2 occupancy and the IGV visualization of MACS ChIP peak and quantitative real-time ChIP PCR assays of these peaks are shown in Fig. 5a, b. The Primer pairs designed across the H1T2 peak regions are given in the supplementary file (Additional file 16: Table S14). IP signals were normalized relative to the signal obtained from the input chromatin and enrichment was calculated as percentage of the input. As can be seen, ChIP PCR assay clearly revealed an enrichment over IgG-negative control for all the ChIP samples analyzed (Fig. 5a). Among these, we have validated 7 representative transcription factor-bound regions including *TBPL1, ING2, JMJD2, HMGB2, SMARCC1, CTCF* and *E2F6* which confirmed the enrichment of H1T2 within the transcription start site regions of these regulatory genes which are either involved in spermatogenesis or embryogenesis [36, 55–58]. H1T2 ChIP signals were also observed at the loci of developmentally important genes including HOX (*HOXB3, HOXB8, HOXC5);* SOX *(SOX12, SOX4, SOX14*); FOX (*FOXB1, FOXG1, FOXC1, FOXE1, FOXK2, FOXJ1, FOXM1*); PAX (*PAXX*) and TBX (*TBX1*) gene clusters, where we observe at least one ChIP-seq peak located within 10 kb of the TSS (Additional file 1: Table S1). Representative genomic regions of the developmentally important loci, FOXB1, FOXC1 and SOX12 were also validated by ChIP PCR using H1T2 immunoprecipitated DNA as the template which confirmed significant enrichment of H1T2 in comparison to IgG immunoprecipitated chromatin control (Fig. 5b). Earlier studies have also demonstrated the nucleosomal retention and bivalent histone marks (H3K4me3/H3K27me3) to be significantly enriched at these loci of developmental importance, including HOX, SOX, FOX, TBX, PAX, CDX, and GATA family transcription factors [33, 59]. All together these results suggest a prominent role of the linker histone H1T2 in organizing specific spermatid chromatin domains to potentially regulate transcription of several developmentally important embryonic genes.

We also validated the occupancy of H1T2 with some representative examples of other classes of genomic loci like LINE elements, intronic, intergenic and CpG islands by ChIP PCR (Fig. 5b). ChIP PCR analysis of the two representative loci of LINE repeat regions confirmed the association of H1T2 to the LINE (intergenic) regions which was the major fraction of genomic loci possessing H1T2 ChIP peaks, with significant enrichment in IP fractions in comparison to IgG pull-down fractions. Being the most successful retrotransposon, LINE elements present an acute challenge to germ cells, which are managed by two repressive mechanisms of piRNA pathway and accumulation of repressive histone PTMs in addition to CpG methylation [31, 60, 61]. LINE-1 (L1) subclass represents some 17–20% of the total human and mouse genomes, which seen to be still accumulating in the genome, as evidenced by the presence of L1 RNA and proteins in germ cells and infrequently in differentiated tissues [62, 63]. Recently it has been observed that human L1 retrotransposition events likely occur in development rather than in the germline, using L1 RNAs transcribed in the embryo and in developing germ cells, suggesting active transcription of L1 in germ cells [64, 65]. Additionally, representative loci of H1T2-bound regions like intron, intergenic and CpG were also validated using specific primers targeting the peak regions compared to a negative control locus devoid of any ChIP signal (Fig. 5b).

### Identification of proteins associated with H1T2-bound genomic regions by IP/MS analysis

**Fig. 6:**
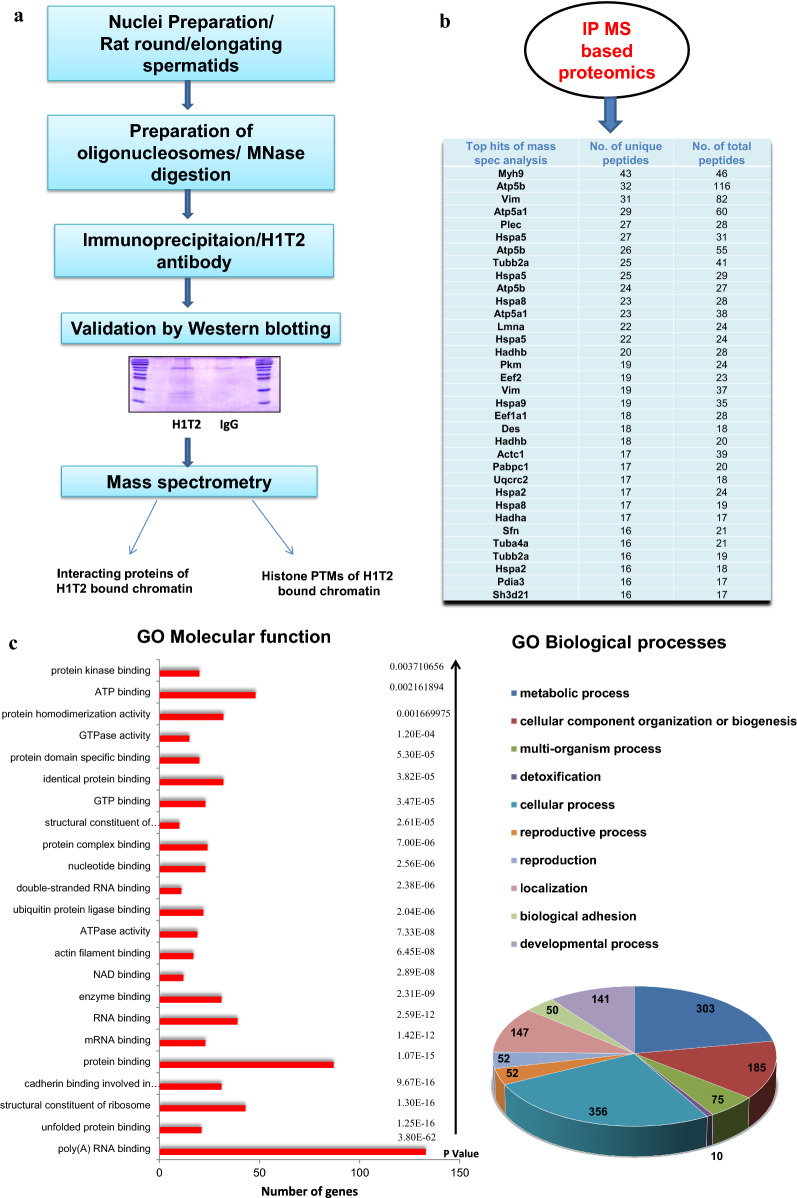

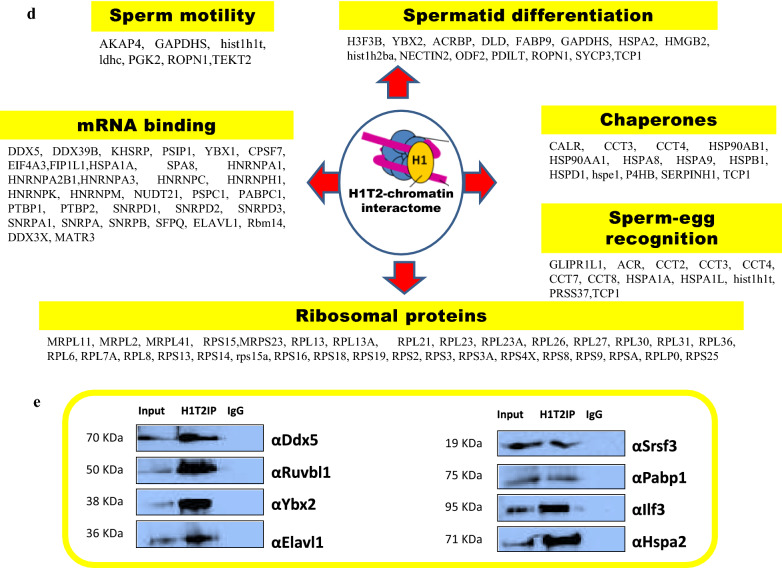
Nucleosome IP/Mass spectrometry analysis for the identification of H1T2-nucleosome interacting proteins. **a** Schematic of the nucleosome IP and mass spectrometry protocol used. **b** Schematic shows the complete list of interacting proteins of H1T2-associated chromatin with top protein hits with more than 15 unique peptides, identified by mass spectrometric analysis of the H1T2 immunoprecipitated chromatin. **c** Gene ontology analysis of the proteins identified in our MS analysis. Diagrammatic representation (bar diagram) of functional classes of H1T2 interacting proteins according to molecular function (P values are given) and pie chart representation of GO biological process associated to the interacting proteins. **d** Different functional classes of H1T2 interacting proteins mainly related to spermatogenesis and sperm function. **e** Validation of proteins associated to H1T2–chromatin identified by mass spectrometry by IP/ WB analysis

To gain further insights into the H1T2 occupied genomic domains, we went ahead to characterize the functional protein complexes associated with H1T2 occupied genomic loci, by subjecting the chromatin IP samples to mass spectrometry analysis as detailed in the flow diagram in Fig. 6a. Analysis of the proteome data identified 425 proteins with ≥ 4 unique peptides, which is usually considered to be significant (Additional file 12: Table S10). We found around 34 proteins with more than 15 unique number of peptides, which includes the proteins of cytoskeleton like actin, tubulin and intermediate filament proteins (plectin, desmin, lamin, vimentin), as listed in Fig. 6b. Cytoskeletal dynamics in spermatogenesis is found to be regulated by the three networks (actin, tubulin and intermediate filament proteins) which function with each other to regulate different cellular processes, such as signaling, cell adhesion, cell motility, maintenance of cell polarity and protein targeting [66–70]. The most significant H1T2–chromatin-associated protein was myosin (Myh9) with 43 unique peptides, which is a reported component of acroplaxome which facilitates acrosomal spreading. Dynein (DYNC1H1) with 13 unique peptides (Additional file 12: Table S10) is also associated with H1T2–chromatin which is a component of manchette microtubules and spermatid nuclear envelope, pointing towards a potential function of H1T2 in nucleo-cytoskeleton interaction during spermiogenesis [14, 71, 72]. Interestingly, H1T2-bound chromatin also interacts with lamin A/C protein encoded by *LMNA* gene, whose function is essential for cytoskeletal dynamics associated with spermatogenesis and its disruption leads to male infertility [73].

Functional annotation of the mass spectrometry identified proteins using DAVID program classified the proteins into the following groups: poly (A) RNA binding (133); ATP-binding (48); structural constituent of ribosome (43); cadherin binding involved in cell–cell adhesion (31); mRNA binding (23); actin filament binding (17); ubiquitin protein ligase binding (22), respectively (Fig. 6c; Additional file 13: Table S11). These groups are summarized in Fig. 6c. An important category of H1T2-bound chromatin interactome was ATP-binding proteins (48 numbers), suggesting a potential role of H1T2 in regulating nuclear shaping involving the energy utilizing functional processes. Notably, ATP synthase subunits (ATP5B, ATP5F1, ATP5C1, ATP5O, ATP5A1, ATP5H) were also observed in this list, whose function is essential for germ cell maturation. Interestingly, their knockdown in Drosophila testes resulted in male infertility and abnormal spermatogenesis [74]. We also carried out functional annotation of H1T2–chromatin interactome on the basis of biological process and the most significant categories are shown in the pie diagram (Fig. 6c; Additional file 14: Table S12). There were 10 groups of proteins: metabolic process (303), cellular component organization or biogenesis (185), multi-organism process (75), detoxification (10), cellular process (356), reproductive process (52), localization (147), biological adhesion (50) and developmental process (141). Further, we also observed the association of the spermatid-specific linker histone H1T2-bound chromatin with a large number of proteins involved in the sperm specific functions like sperm motility, spermatid differentiation and sperm-egg recognition (Fig. 6d; Additional file 15: Table S13).

Male infertility has also been noted upon the ablation of several genes encoding RBPs [75]. Indeed, RNA-binding proteins were one of the major classes of proteins associated to H1T2-bound chromatin in spermatids (Fig. 6c; Additional file 13: Table S11). The major function attributed to spermatid-specific RBPs is the post‐transcriptional regulation of mRNAs which are highly expressed in germ cells [75]. Specifically, the mRNPs function in the storage of silenced transcripts until factors in the elongation phase of spermiogenesis trigger their translation to support final steps in spermatozoan development and fertilization [76–78]. The mRNA-binding proteins interacting with H1T2-bound chromatin are listed in Fig. 6d. Numerous RBPs are synthesized solely in late phases of spermiogenesis, ensuring a temporal regulation of their target mRNAs [79]. Embryonic Lethal Abnormal Vision (ELAV) L1 is one such candidate RBP pulled down by H1T2 antibody which is reported as a key regulator of posttranscriptional regulation in spermatocytes, whose targeted deletion results in male sterility phenotype [80]. More recent studies have supported the mRNA regulatory role of Elavl1 by specifically associating to *Tnp* mRNAs to control the delayed timing of their translation [81]. H1T2–chromatin IP/MS analysis also identified YbX2, another potential mRNA repressor involved in the regulation of translation whose knockout resulted in male infertility and azoospermia condition [82]. Notably we also found Ddx5 in our list of H1T2–chromatin interacting proteins, which is considered to be an essential protein for both transcriptional and posttranscriptional roles in the maintenance and function of spermatogonia [83]. PABP1 was the other important protein pulled down with H1T2–chromatin, whose function has been extensively characterized in germ cells and shown to have important roles in mRNA stability and translation [84]. Other candidate RBPs includes PSPC1, PTBP1, PTBP2, SFPQ, RBM14, SNRPs and HNRNPs. We went ahead to further validate the association of a representative set of proteins with H1T2 bound chromatin by western blot analysis (Fig. 6e). More recently, LINE repeats have been reported to harbor repressive RBPs thereby contributing to the evolution of new, lineage-specific transcripts in mammals [85]. In this context, we did observe a substantial overlap between the H1T2–chromatin interacting proteins and LINE1 ORF1 interactome which function in the regulation of retrotransposons [85, 86]. It is worth noting here that LINE Rn_L1 subclass of LINE elements that we have observed to be associated with H1T2, is indeed an active element containing ORF1 and ORF2 within the repeat [32].

### Identification of PTMs of histones associated with H1T2 occupied chromatin domain

**Fig. 7:**
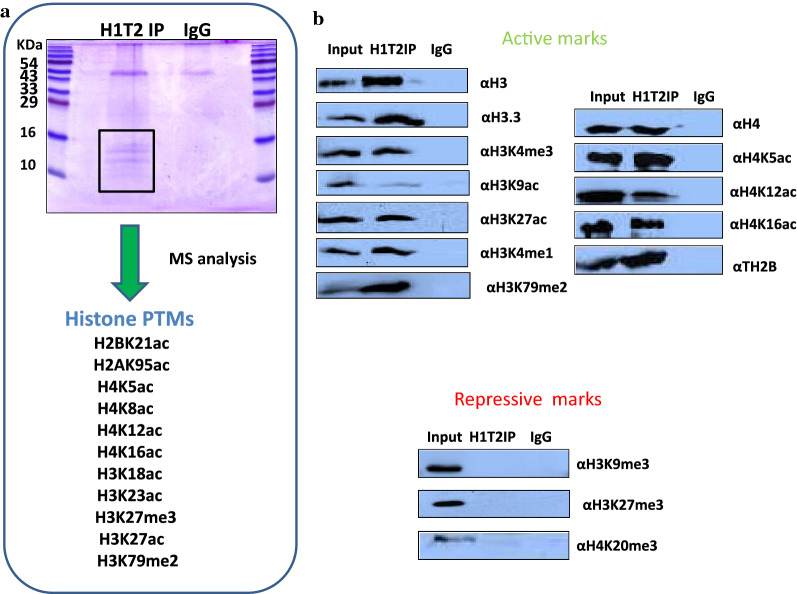
Identification of histone PTMs associated to H1T2 bound chromatin. **a** Coomassie stained gel of the acid-soluble fractions of H1T2 and IgG immunoprecipitated chromatin samples and the list of histone PTMs identified by MS analysis. **b** IP/WB analysis of the histone PTMs associated to H1T2 bound chromatin. Western analysis with antibodies against active histone PTMs gave positive signal upon immunoprecipitation with H1T2 antibody, whereas H1T2 bound chromatin was found to be devoid of any repressive modifications

To investigate the epigenetic status of H1T2-associated chromatin in terms of histone PTMs, acid-soluble fraction was prepared from H1T2 immunoprecipitated chromatin and subjected to mass spectrometry analysis. Histone PTMs associated to these H1T2 bound chromatin loci were identified to be acetylation marks like H2BK21ac, H2AK95ac, H4K5ac, H4K8ac, H4K12ac, H4K16ac, H3K18ac, H3K23ac and H3K27ac and methylation marks like H3K27me2 and H3K79me2 (Fig. 7a). Western blot analysis confirmed a combination of acetylated H4 peptides (H4K5ac, H4K12ac, and H4K16ac) which are activation marks to be highly enriched in the immunoprecipitated chromatin, whereas no signals were observed in IgG control samples (Fig. 7b). Recent evidences have shown the involvement of H1T2 in the initiation of chromatin remodeling in spermatogenesis wherein H4 acetylation acts as a key to open up the chromatin in the doughnut structure in the apical region to facilitate the histone replacement in round spermatids [7]. Another study has also revealed the enrichment of BRD4, H3K9ac, and H4K5ac, H4K8ac, H4K12ac, and H4K16ac towards TSSs of active genes in round spermatids, and their enrichment correlates with transcription levels [87]. Further analysis also revealed the enrichment of other active histone PTMs like H3K4me3, H3K9ac, H3K27ac, H3K4me1 and H3K79me2, whereas the repressive modifications like H4K20me3, H3K9me3, H3K27me3 were absent in the H1T2 immunoprecipitated chromatin fraction (Fig. 7b). Incorporation of H1T2 in early round spermatids can be considered as an additional player in the chromatin reorganization process in addition to H3K79 methylation accompanying H4 acetylation as has been reported previously [88, 89]. Notably, we also found H1T2 occupied chromatin domains to harbor the histone variants like H3.3 and TH2B as demonstrated by IP/western blot. These core histone variants have been shown to be involved in the formation of transitional subnucleosomal structures during histone to transition proteins/protamine transition [90–93]. Taken together, the results presented so far suggests a unique property to the spermatid-specific H1, H1T2 to organize the polar domain of the spermatid genome by clustering the actively transcribed or to be transcribed genomic domains and in association with extensive number of chromatin interacting partners and cytoskeleton mediated mechanical force. H1T2 may therefore contribute to the chromatin remodeling process as well as nuclear shaping event that occurs during the elongation of spermatid nucleus in rodents.

## Discussion

Linker histone H1 was thought to be a chromatin organizer protein that is primarily involved in generation and stabilization of higher order structure of eukaryotic chromatin. But more recent studies have shown that the somatic subtypes as well as its tissue specific variants have many genomic functions beyond the architectural role [94, 95]. Mammalian testes express three linker histone variants, histone H1t, H1T2 and HILS1 in a stage-specific manner. As mentioned earlier, gene knockout studies have shown that H1T2 is an essential gene for spermiogenesis and these mice show an infertile phenotype [23]. A more interesting finding was the distinct localization pattern of the histone in the apical region of the spermatid nucleus showing a progressive change in the localization pattern and finally disappearing from chromatin during the final stages of spermiogenesis. H1T2 mutant mice also showed an abnormal chromatin condensation pattern with a defect in histone-to-protamine replacement process [22, 23]. With this background available in the literature, we initiated the present study to identify and characterize the genomic chromatin domains that are occupied by histone H1T2. For a meaningful analysis of the genome-wide occupancy analysis of histone H1T2, it is essential that the antibody used is of high specificity and does not cross-react with other somatic as well as testes-specific histone H1 subtypes. All the validation experiments carried out on the authenticity and specificity of the anti-histone H1T2 antibodies that we have generated show that it is highly specific for H1T2, including the ChIP experiment. Furthermore, our immunofluorescence experiments also showed the typical polar localization pattern in the apical region of spermatids as has been reported earlier in the literature (Fig. 2).

The results of our ChIP-seq analysis of H1T2 have shown a predominant occupancy at intergenic regions (71.56%), in addition, a significant number of reads was observed in genic regions [promoters (2.94%) and exons (2.43%) and introns (19.27%)] (Fig. 3d). A more in-depth analysis of the promoter-occupied regions showed a perfect overlap with TSS region of approximately 321 genes. Most of the genes belonged to the class of spermatogenesis related functions, including many germ cell-specific transcription factors like *TBPL1*, *ING2* etc. and also embryonic transcription factor like *HOXC5*. A surprising finding was the occupancy of H1T2 at the TSS site of *CTCF*, a global chromatin organizer that is involved in the enhancer-promoter interaction regulating the 3D-architecture of chromatin. It may be pertinent to point here that the spermatid genome is transcriptionally active at the stages between round and elongating spermatid stages [9, 96], during which time histone H1T2 makes its appearance. The association of H1T2 with transcriptionally active spermatid chromatin was also corroborated with the association of most of the histone PTM signatures characteristic of active chromatin. In addition, histone H4K12Ac mark was also associated with these domains as revealed by IP/MS and IP/WB analysis (Fig. 7). This histone mark is prominent in the post acrosomal region of the spermatids and is also preferentially distributed in the promoter region of many genes relevant for early embryonic development [97]. Additionally, we also found the association of H3K79 methylation with these chromatin domains, which has shown to be a conserved feature of spermatid chromatin preceding the process of histone-to-protamine transition which is in agreement with the role of H1T2/H1FNT in this process (Fig. 7) [88, 89]. These chromatin domains were also enriched with two core histone variants H3.3 and TH2B which are known to destabilize the nucleosome particle [93, 98]. Most importantly, the repressive marks like H3K9, K27 and HK20 methylation were completely absent in these domains, thus strengthening the argument that histone H1T2 is associated with open transcriptionally active chromatin domain in the spermatid nucleus.

Polar localization of H1T2 is considered as a molecular signature for the chromatin organization in spermatids since there is a delayed nuclear condensation and morphological restructuring of sperm in H1T2 disrupted mutant mice [22, 23]. It is also conjectured that this linker histone variant is linked to nuclear shaping during the elongation of spermatids, which is another major morphological event during this particular stage of spermiogenesis [99]. In this context, our identification of many of the proteins belonging to the class of nucleoskeleton proteins to be associated with the H1T2 occupied chromatin gains significance suggesting that these chromatin domains are tethered to the nucleoskeleton structure during the spermatid elongation process. During nuclear shaping process, component of nuclear lamina play significant role, and it is very interesting that lamin A/C, a structural component of the acroplaxome [71], is one of the very significant proteins picked up in our ChIP/MS analysis. Lamin A/C is involved in the nucleocytoskeletal force transmission and essential for acrosome biogenesis and spermatid head shaping [73]. Furthermore the identification of tubulin, actin and intermediate filament proteins in the proteome of H1T2 bound chromatin (Fig. 6b), suggest the possibility of H1T2 not only essential for the maintenance of polarity in spermatids, but also involved in connecting the spermatid chromatin to the cytoskeleton in the sperm head and further confirms the inevitability of polar distribution of H1T2. It is very likely that the inherent property of H1T2 to be involved in this nuclear shaping process might be contributed by the highly extended C-terminus domain containing SR repeats, ATP-binding Walker motif and the putative coiled–coil domains (Fig. 1b). As mentioned earlier, similar domain architecture is present in Drosophila Mst77f protein, whose function is crucial for sperm development and nuclear shaping [9, 28]. Notably, all these domains in the extended CTD are absent in human H1T2 protein, which can be correlated with the existing observation of considerable divergence of H1 CTD throughout evolution accounting for the bulk of sequence heterogeneity between H1 variants [100]. It would be an interesting area of research to understand whether the sequence divergence of sperm specific proteins involved in the chromatin remodeling along with the interacting partners can also be determinants of considerable diversity in sperm morphology [101].

Recently, several epigenomic studies have shown that DNA modification status, chromatin proteins and associated histone marks, as well as sperm derived RNA are passed on to the embryo, any disruption of which can lead to male infertility, abnormal embryo development and/or trans-generational inheritance [102]. Many studies have also been focused on the role of sperm derived RNAs in embryonic development [103–105], of which one study identified ~ 18,000 mRNA are delivered to the embryo. H1T2 ChIP peaks were enriched in some of the identified genes like *AKAP4*, *FOXG1*, *CLUSTERIN* and *WNT5a* genes whose mRNAs participate in the male pro-nucleus formation and development (Additional file 1: Table S1) [104]. In addition, mature spermatozoa are identified as a source of LINE-1 RNA populations which are immediately available for retro transcription early after fertilization [104–107]. Majority of the H1T2 bound genomic region was identified as LINE L1 (5480 peaks; Fig. 3e), possibly embedded in the active chromatin domains. It is interesting to note that full length L1 transcripts are highly expressed in germ cells, especially ORF1 expression is well reported in mouse/rat spermatids [108, 109]. In addition, LINE L1 elements have been reported to be under-methylated and histone retained, specifically in the nuclear periphery where the pronuclear reverse transcription occurs in early development [107, 110–112]. We found many LINE L1 specific RBPs including MATR3, ELAVL1, PTBP1 and HNRPs in our H1T2 IP/MS analysis listed proteins, which are considered as repressive RBPs which repress splicing events and 3′ end processing within and around LINEs, thereby contributing to the evolution of new, lineage-specific transcripts in mammals (Fig. 6d) [85]. It is of considerable interest that many of the proteins that co-IP with H1T2 bound chromatin, can also be identified in the L1 ORF1 and its ribonucleoprotein interactome identified by mass spectrometry in a recent study [86]. IP/WB analysis also validated several of proteins involved in splicing, mRNA export, and transcript stability like Ddx5, Ybx2, Elavl1, Pabp1 and Ilf3, known to be associated with transcriptionally active chromatin domain. In addition, we also observed Srsf3 and Ruvbl1 in our proteome analysis which have been implicated recently as sperm-carried molecular factors involved in oocyte fertilization and embryo growth (Fig. 6e) [113].

**Fig. 8:**
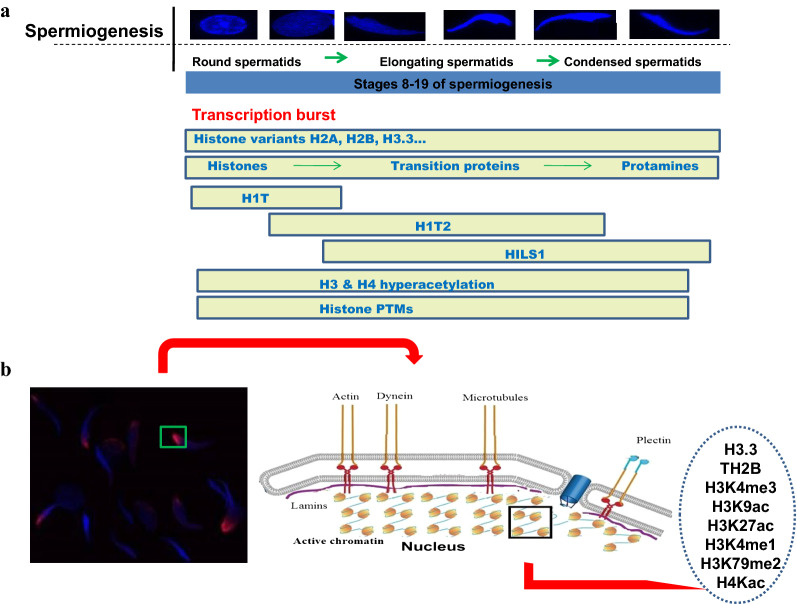
Schematic representation of the process of spermiogenesis and chromatin architecture of H1T2 occupied polar apical region of spermatids. **a** Spermiogenesis is a lengthy post-meiotic developmental process of spermatogenesis in which the round spermatids undergo several morphological and biochemical modifications to form the mature spermatozoa. Substantial remodeling of chromatin during spermiogenesis involves the replacement of standard histones with histone variants (H1t, H1T2, HILS1, H3.3A, and H3.3B) and testes-specific histones (TH2A, TH2B, and TH3) that should favor an open chromatin conformation. Hyperacetylation of H3 and H4 histones probably facilitates the substantial and continuous repackaging of DNA with transition proteins, further replaced by protamines. Leaky transcription of the spermatid genome is considered as a functional consequence of chromatin remodeling in spermatids [121]. **b** A schematic model showing the involvement of H1T2 in organizing the nucleoskeleton associated active chromatin architecture of the polar apical region of spermatids

The process of spermatogenesis is a very complex, developmental and differentiation process involving several facets of remodeling that is influenced by the testes-specific core histone and linker histone variants which appear in a definite and stage-specific manner (Fig. 8a). As mentioned earlier, the three mammalian testes-specific linker histone variants, namely H1t, H1T2 and HILS1 appear in a sequential and stage-specific manner during spermatogenesis in the testes with an overlapping presence (H1t-H1T2 and H1T2-HILS1) having unique chromatin organization property and putative specialized genomic function. We have recently delineated the genomic functions of H1t and HILS1 [24, 25], which revealed the preferential association of these two H1s to the repressed chromatin loci of spermatocytes and spermatids respectively. The results presented in this investigation have clearly shown the unique property of H1T2, primarily occupying genomic regions which are actively transcribed during the elongating stage of spermiogenesis probably facilitating the process of transcription of many spermatogenesis related genes and also few of the genes relevant for early developmental process. This observation may look surprising since histone H1 is normally associated with repressed chromatin domains. However, there are some examples in the literature wherein multiple functions have been attributed to histone H1 not simply as chromatin architectural protein, by functioning as a part of multi-protein complexes [94, 95]. The open chromatin structure of H1T2 occupied polar spermatid genome may also serve as a prerequisite to initiate the global chromatin remodeling process involving histone to transition proteins/protamine transition. Another important observation of this study is the association of the H1T2-occupied spermatid chromatin with many of the nucleo-skeleton components, which strongly suggests the possibility that this tethering event is possibly related to the nuclear shaping process which is a characteristic feature of the elongation of spermatid nucleus. We have summarized our findings in the schematic diagram depicted in Fig. 8b, in the context of events that characterize the major chromatin remodeling process that occur during mammalian spermiogenesis.

## Conclusions

Histone H1T2 is the most divergent among the three testes-specific histone H1s containing several important domains like SR domains, Walker motif, and coiled-coil domains. The three testes-specific linker histone variants differ considerably in the N-terminal and C-terminal domain sequences. The C-terminus in particular is the most divergent and it will be challenging to address the role of these sequence variations among these linker histone variants in facilitating the necessary chromatin remodeling events during spermatogenesis. It would be very interesting to see the function of these individual sub-domains in many of the chromatin-associated functions. Surprisingly, many of these sub-domains are absent in human H1T2, the significance of which will be an intriguing question to address. In conclusion, the present study has identified the genomic domains occupied by histone H1T2 and characterized many of the chromatin features in terms of associated proteins and the core histone modifications, which should form the basis of future investigations in delineating the molecular and cellular mechanisms of global chromatin remodeling that is a unique and characteristic feature of spermatogenesis.

## Methods

### Analysis of H1T2 sequence conservation and protein structure

Amino acid alignments were performed online using Clustal X, a graphical interface for the ClustalW multiple sequence alignment program of Jalview (http://www.jalview.org). Prediction of coiled-coil structures was predicted using the online tool (https://embnet.vital-it.ch/cgi-bin/COILS_form_parser) [114, 115]. Secondary structure of rat H1T2 sequence was predicted by Phyre2 [116].

### Antibody generation

To generate antibody against the CTD of rat H1T2 protein, rabbits were immunized with synthetic peptide, EQQYVSAKEQEYVRTKEQEC and a 12 weeks protocol was used for the generation of the antibody. The custom made antibody from the Abgenex Company (Bhubaneshwar, India) was used for the present study.

### Extraction and purification of histones from rat tissues

Rat testes (35–50 days old) were decapsulated and homogenized in 5 volumes of cell lysis buffer (10 mM Tris–HCl, pH 7.4, 10 mM NaCl, 3 mM MgCl_2_, 0.1% Nonidet P-40, 1 mM PMSF, 10 mM NaHSO3, 10 mM sodium butyrate, protease inhibitor mixture, phosphatase inhibitor mixture). The homogenate was filtered through four layers of bandage cloth and centrifuged at 1500 × *g* for 10 min at 4 °C. Histones were extracted from the pellet of crude nuclei by suspending purified nuclei in 0.4 N H_2_SO_4_ as prescribed previously [117], homogenized and incubated on ice for 30 min. The sample was centrifuged for 10 min; 8000 *g* at 4 °C to pellet the residual chromatin. The extracted proteins were precipitated at 4 °C from the supernatant by making it to 30% with respect to TCA. After keeping on ice for 30 min, the proteins were recovered by centrifugation. The pellet of proteins so obtained was sequentially washed with cold acetone containing 0.05% HCl and ice cold acetone. The pellet recovered was dried, dissolved in water and stored in aliquot at − 20 °C.

### Dot-blot analysis

Two microgram of the synthetic peptide (303-EQQYVSAKEQEYVRTKEQEC-321) was spotted on the nitrocellulose membrane, dried and subjected to western blot analysis using the anti-H1T2 antibody.

### Western blot analysis

The proteins resolved by SDS-PAGE were electrophoretically transferred onto nitrocellulose membranes presoaked in transfer buffer, using a semidry transfer technique. After the transfer, the membrane was blocked using the wash buffer, PBST (1 × PBS with 0.05% Tween 20), containing 5% BSA or milk powder for 1 h at room temperature. It was then washed with the same buffer lacking BSA or milk powder. A suitable dilution of the primary antibody was added in PBST supplemented with 1% BSA or milk powder and incubated for overnight on a rocking platform. The unbound antibody was removed by washing the membrane thrice for 5 min each using wash buffer. A suitable dilution of an appropriate secondary antibody (anti-rabbit/anti-mouse IgG) conjugated to horse radish peroxidase (HRP) in PBST with 1% BSA or milk powder was added and incubated for 1 h. After the antibody incubation, the membrane was washed thrice with the wash buffer for 5 min each. The membrane was placed in the chemiluminescence substrate solution and incubated for 2 min and developed using the ECL kit (Thermo Scientific). For the peptide competition assay, 50-fold molar excess of the peptide were added to the antibody solution and mixed for 3 h at 4 °C before the addition to the blot. The source of the antibodies is given in Table 1.

**Table 1:** List of antibodies used in the present study

Antibodies	Host	Company name	Cat number	Applications
Ddx5	Rabbit	Abcam	ab126730	WB
Ruvbl1	Rabbit	Abcam	ab133513	WB
Ybx2	Rabbit	Abcam	ab154829	WB
Elavl1	Rabbit	Abcam	ab200342	WB
Srsf3	Rabbit	Abcam	ab198291	WB
Pabp1	Rabbit	Abcam	ab21060	WB
Ilf3	Rabbit	Abcam	ab92355	WB
Hspa2	Rabbit	Invitrogen	PA5-81895	WB
H3	Rabbit	Abcam	ab1791	WB
H3.3	Rabbit	Abcam	ab62642	WB
H3K4me3	Rabbit	Abcam	ab8895	WB
H3K9ac	Rabbit	Abcam	ab4441	WB
H3K27ac	Rabbit	Abcam	ab4729	WB
H3K4me1	Rabbit	Abcam	ab8895	WB
H3K79me2	Rabbit	Abcam	ab177184	WB
H4	Rabbit	Abcam	ab10158	WB
H4K5ac	Rabbit	Abcam	ab51997	WB
H4K12ac	Rabbit	Abcam	ab177793	WB
H4K16ac	Rabbit	Abcam	ab109463	WB
H3K9me3	Rabbit	Abcam	ab8898	WB
H4K20me3	Rabbit	Abcam	ab9053	WB
H3K27me3	Rabbit	Abcam	ab192985	WB

*WB* western blotting

### Decondensation and immunofluorescence of testicular cells

Rat testes were decapsulated and total testicular cells were decondensed by resuspending in decondensation buffer (PBS, 10 mM dithiothreitol) and incubated at 4 °C for 1 h in an end to end rotor (Kolthur et al. 2009). Cells obtained after centrifuging at 1500 *g* for 10 min at 4 °C, were washed with PBS and fixed with 4% paraformaldehyde (in PBS) and permeabilized with 0.1% Triton X-100 (in PBS) for 15 min. Non-specific sites were blocked with 1% BSA in PBS. Smears were incubated with selected primary antibodies, anti-H1T2 and counterstained with corresponding secondary antibodies conjugated with Alexa fluor 488 or Alexa Fluor568. Nuclei were stained with DAPI (Sigma) and images were acquired and analyzed in a Zeiss confocal laser scanning (LSM 510 META, Carl Zeiss) microscope.

### ChIP-sequencing analysis of H1T2-bound chromatin and data analysis

Chromatin immunoprecipitation (ChIP) was carried out using the published protocol [118]. Briefly, the nuclei were prepared from 35–50 days postnatal rat testes and decondensed using 10 mM DTT, and were fixed using 1% formaldehyde for 10 min at room temperature, quenched with 125 mM glycine for 5 min, harvested and sonicated to generate fragments of 100–300 bp. For ChIP, 25 μg of sonicated chromatin was immunoprecipitated overnight with 5 μg anti-H1T2 antibodies. Immunocomplexes were captured on 50 μl protein A Dynabeads. Beads with bound immunocomplexes were washed, eluted and reverse cross-linked at 65 °C overnight, and DNA was extracted by phenol–chloroform method. Input was prepared with 5% of the sonicated chromatin.

The input and ChIP DNA libraries were prepared using the NEBNext Ultra II DNA library preparation kit. Sequencing was performed with Illumina HiSeq X Ten system using paired-end reads of 150-nt length. Sequenced reads containing adaptor sequences were trimmed out and filtered with Phred quality threshold as 20. Read pairs falling below 20 bp after clipping were removed. Clean reads were uniquely aligned against UCSC *Rattus norvegicus* genome (rn6) using Bowtie2 (version 2.2.3) with “no mixed”, “-very sensitive” and “no discordant” parameters. All aligned files were then made free from PCR duplicates using samtools. Replicates of both the control and treated were merged, respectively, into a single BAM file. Thereafter, the merged BAM file of the input samples and the merged BAM file of treated samples were used for the subsequent peak calling by MACS1.4.2 with p-value cutoff of 1e-05. Read enrichments/peaks were first called for each replicate with the uniquely mapped reads by MACS1.4.2 with default parameters except the effective genome size corrected for rn6. Chromosome-wise distribution plots were generated using custom R script. H1T2 peak distribution was identified at genomic locations enriched in CpG islands, repetitive elements, exons, introns, intergenic regions, 3′UTR and 5′UTR using HOMER v4.7 (for annotation). Further subclassification of LINE L1 repeat elements was made using the repeat masker data of UCSC table browser. Box plots were created using the R 3.3.2 statistical package. De novo motif identification was done using 300 bp regions surrounding overlapping peak summits as target set and shuffled set of same overlap sequences as background set with MEME software.

### ChIP PCR analysis

ChIP chromatin was prepared as described above. Beads with bound immunocomplexes were washed, eluted, and reverse cross-linked at 65^0^C overnight, subjected for RNase (final; 40 μg/ml) and proteinase K (Final; 100 μg/ ml) treatment and DNA were extracted by phenol–chloroform method. Input was prepared from 5% of the sonicated chromatin. 2 μL input or ChIP sample from the recovered DNA suspension of 50 μL was used for performing the qPCR analysis. Specific primers were designed against the HIT2 peaks obtained from ChIP-sequencing experiment (primers are listed in Additional file 16: Table S14). The enrichment of specific regions of the genome were validated by qPCR reaction using ChIP DNA (20 ng), 300 nM forward/reverse primers using SYBR kit from TAKARA. The *C*t values of the duplicates showed minimal variability, and *C*t values were used for calculating the percent of input using the following formula:

$${\text{Percent}}\,{\text{input}} = 100 \times 2({\text{zdjusted}}\,{\text{input}} - {\text{Ct}}({\text{IP}})).$$

### MNase digestion and chromatin immunoprecipitation

MNase immunoprecipitation was done according to the previous method [93]. Briefly, the rat testes were dissected and lysed in the Lysis buffer (Tris–HCl pH 7.4 15 mM, KCl 60 mM, NaCl 15 mM, sucrose 0.34 M, EDTA 2 mM, EGTA 0.5 mM, spermidine 0.65 mM, dithiothreitol 1 mM, Triton X-100 0.03%, glycerol 1%, Protease Cocktail Inhibitor) followed by centrifugation for 15 min at 200 × *g* at 4 °C. Nuclei were suspended in wash buffer (Tris–HCl pH 7.4 15 mM, KCl 60 mM, NaCl 15 mM, sucrose 0.34 M, dithiothreitol 1 mM, Protease Cocktail Inhibitor) and directly proceeded for MNase digestion by suspending the nuclei in MNase buffer (Tris–HCl pH 7.5 10 mM, KCl 10 mM, CaCl_2_ 2 mM). Sperm nuclei were digested with MNase in MNase buffer for 5 min 37 °C. Reaction was stopped with the addition of 10 mM EDTA on ice. The soluble nucleosome fraction was prepared by centrifugation at 650 × *g* for 10 min at 4 °C, mixed with LSDB250 buffer (20% glycerol, 50 mM HEPES, 3 mM MgCl_2_, 250 mM KCl, protease and phosphatase inhibitor cocktail) and proceeded to immunoprecipitation protocol.

### Mass spectrometry for identification of H1T2-bound chromatin-associated proteins and histone post-translational modifications

The oligonucleosomes fraction prepared by MNase digestion was incubated with either anti H1T2 antibody or pre-immune IgG for overnight at 4 °C in a rotating platform. Protein a/G dynabeads were added on the next day and LSDB250 buffer was used as the wash buffer for the MNase-IP studies. Proteins bound to the beads were extracted using the elution buffer from the Pierce Co-IP kit. The eluted protein complexes were resolved on a 15% SDS gel and run for about 1 cm, then the gel was subjected to Coomassie staining. The Coomassie stained wells corresponding to IP and IgG lanes were excised and subjected to downstream analysis. To determine the proteins associated to H1T2-bound chromatin, we have outsourced the samples for LC–MS/MS analysis which was carried out at the Taplin Biological Mass Spectrometry Facility, Cell Biology Department, Harvard Medical School.

Excised gel bands were cut into approximately 1 mm^3^ pieces. As described previously [98], gel pieces were then subjected to a modified in-gel trypsin digestion procedure [119]. Gel pieces were washed and dehydrated with acetonitrile for 10 min. followed by removal of acetonitrile. Pieces were then completely dried in a speed-vac. Rehydration of the gel pieces was with 50 mM ammonium bicarbonate solution containing 12.5 ng/µl modified sequencing-grade trypsin (Promega, Madison, WI) at 4 °C. After 45 min, the excess trypsin solution was removed and replaced with 50 mM ammonium bicarbonate solution to just cover the gel pieces. Samples were then placed in a 37 °C room overnight. Peptides were later extracted by removing the ammonium bicarbonate solution, followed by one wash with a solution containing 50% acetonitrile and 1% formic acid. The extracts were then dried in a speed-vac (~ 1 h). The samples were then stored at 4 °C until analysis. On the day of analysis the samples were reconstituted in 5–10 µl of HPLC solvent A (2.5% acetonitrile, 0.1% formic acid). A nano-scale reverse-phase HPLC capillary column was created by packing 2.6 µm C18 spherical silica beads into a fused silica capillary (100 µm inner diameter ×  ~ 30 cm length) with a flame-drawn tip (Peng et al. 2001). After equilibrating the column each sample was loaded via a Famos auto sampler (LC Packings, San Francisco CA) onto the column. A gradient was formed and peptides were eluted with increasing concentrations of solvent B (97.5% acetonitrile, 0.1% formic acid). As peptides eluted they were subjected to electrospray ionization and then entered into an LTQ Orbitrap Velos Pro ion-trap mass spectrometer (Thermo Fisher Scientific, Waltham, MA). Peptides were detected, isolated, and fragmented to produce a tandem mass spectrum of specific fragment ions for each peptide. Peptide sequences (and hence protein identity) were determined by matching protein databases with the acquired fragmentation pattern by the software program, Sequest (Thermo Fisher Scientific, Waltham, MA) [120]. All databases include a reversed version of all the sequences and the data were filtered to between a one and two percent peptide false discovery rate. The complete list of H1T2-associated list of proteins with more than 3 unique peptides is given in the table Additional file 11: Table S9.

For the mass spectrometric analysis of core histone PTMs in the immunoprecipitated chromatin complex, the final eluate was subjected to histone extraction using 0.4 n H_2_SO_4_. After precipitation of histone proteins with 30% trichloroacetic acid, the histone extracts were loaded onto a 12% SDS gel and resolved completely. 5–25 kDa portion that contained all core histones were excised from each gel (H1T2 IP and IgG IP lanes) and sent for LC–MS/MS analysis of the histones (PTMs).

## Supplementary information

**Additional file 1: Table S1.** H1T2 ChIP-seq data: MACS 1.4.2 output file with annotations. H1T2 ChIP peaks MACS 1.4.2 with p-value cutoff of 1e-05. A total of 11,570 peaks were identified as overlapping peaks between the two replicates and the H1T2 peaks were annotated using HOMER v4.7.

**Additional file 2: Table S2.** Chromosome-wise distribution of H1T2 peaks. Representative excel file of the number of H1T2 peaks across different chromosomes.

**Additional file 3: Figure S1.** Chromosome-wise peak length distribution of H1T2 ChIP peaks. Box plot representing the H1T2 associated average peak length (*y*-axis) across different chromosomes (*x*-axis).

**Additional file 4: Table S3.** Representative excel file of the peak length of H1T2 peaks across the chromosomes.

**Additional file 5: Figure S2.** Fold enrichment of H1T2 peaks. Box plot showing the fold enrichment of H1T2 peaks (*y*-axis) across different rat chromosomes.

**Additional file 6: Table S4.** Representative excel file of the fold enrichment of H1T2 peaks across chromosomes.

**Additional file 7: Table S5.** Repeat elements associated with H1T2. Excel file representing the different classes of repeat elements associated with H1T2.

**Additional file 8: Table S6.** LINE elements subclasses associated with H1T2. Representative excel file of the subclasses of LINE repeats associated with H1T2.

**Additional file 9: Table S7.** Motif identification. Table represents the significant motifs identified from the overlapping peak summits using MEME analysis.

**Additional file 10: Table S8.** GO enrichment analysis of the genes whose promoter-TSS is occupied by H1T2. Excel file related to Fig. 4b: GO analysis_DAVID_Biological processes.

**Additional file 11: Table S9.** GO enrichment analysis of the genes whose promoter-TSS is occupied by H1T2. Excel file related to Fig. 4c: GO analysis_DAVID_Molecular function.

**Additional file 12: Table S10.** H1T2 IP/MS analysis. List of H1T2 associated protein identified by mass spectrometric analysis (≥ 4 number of unique peptides).

**Additional file 13: Table S11.** GO enrichment analysis of the H1T2 IP/MS identified proteins. Excel file related to Fig. 6c: GO analysis_DAVID_ Molecular function.

**Additional file 14: Table S12.** GO enrichment analysis of the H1T2 IP/MS identified proteins. Excel file related to Fig. 6c: GO analysis_DAVID_ Biological processes.

**Additional file 15: Table S13.** GO enrichment analysis of the H1T2 IP/MS identified proteins. Excel file related to Fig. 6c: GO analysis_DAVID_ Sperm related functions.

**Additional file 16: Table S14.** List of primers used for H1T2 ChIP-PCR analysis.

## Data Availability

The data sets supporting the conclusions of this article are submitted to Gene Expression Omnibus repository (GSE162144).
