# Supplementary figures and images for: Genome-wide occupancy reveals the localization of H1T2 (H1fnt) to repeat regions and a subset of transcriptionally active chromatin domains in rat spermatids
Source: Epigenetics Chromatin. 2021 Jan 6;14:3. doi: 10.1186/s13072-020-00376-2 (PMC7788777; doi:10.1186/s13072-020-00376-2)

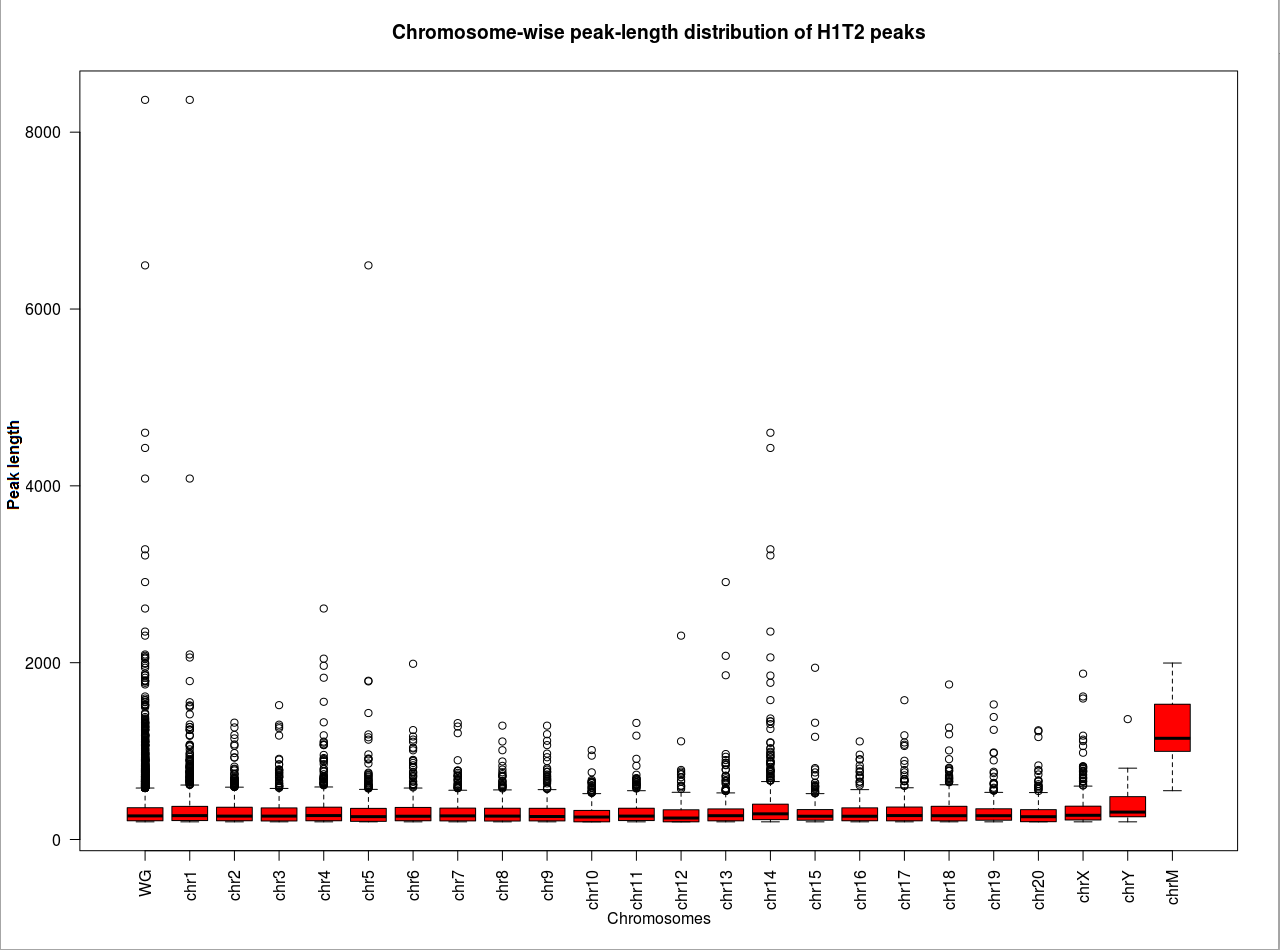

Supplement: Supplementary file 3 — Additional file 3: Figure S1. Chromosome-wise peak length distribution of H1T2 ChIP peaks. Box plot representing the H1T2 associated average peak length (y-axis) across different chromosomes (x-axis). [file 13072_2020_376_MOESM3_ESM.png]

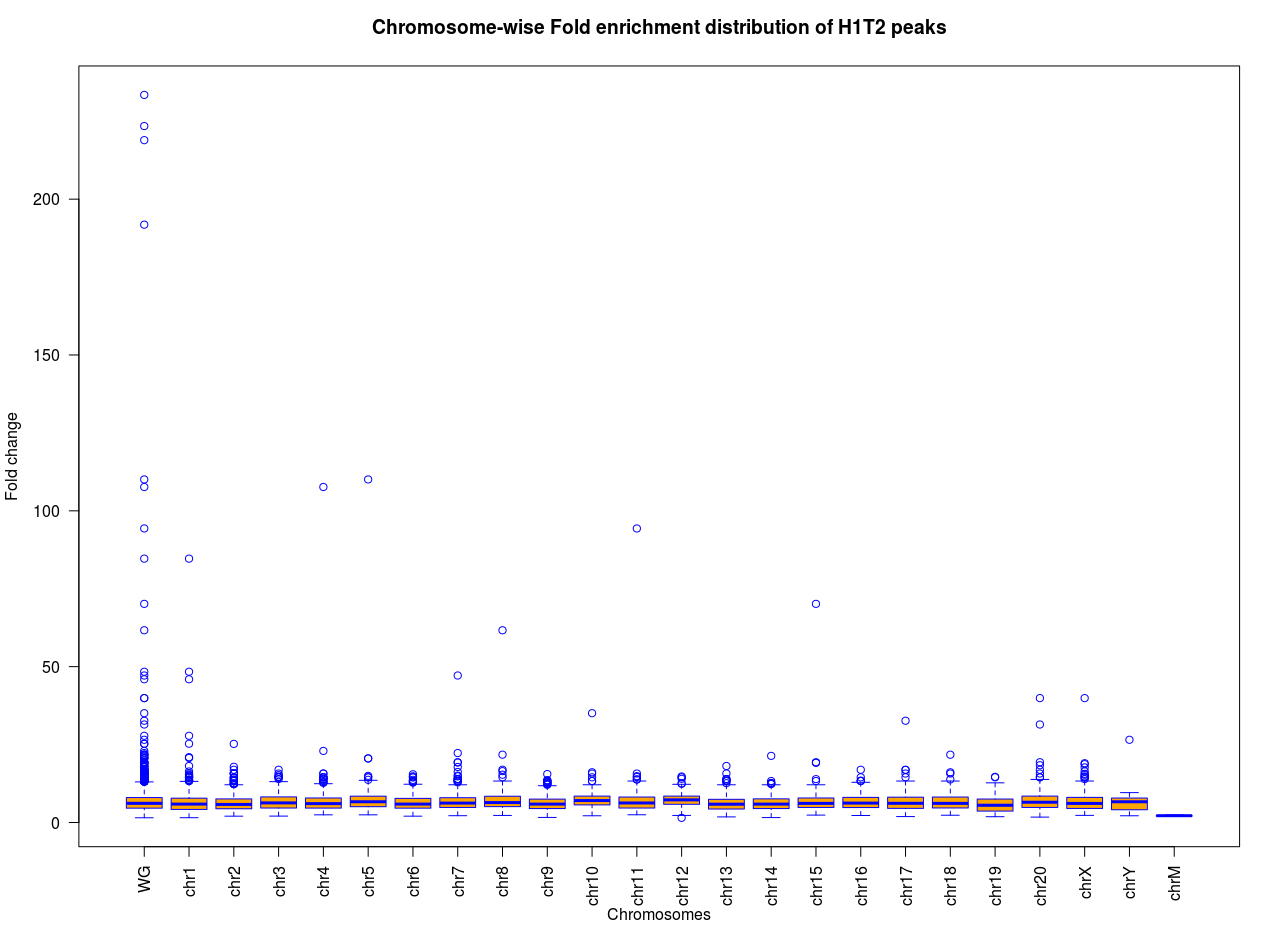

Supplement: Supplementary file 5 — Additional file 5: Figure S2. Fold enrichment of H1T2 peaks. Box plot showing the fold enrichment of H1T2 peaks (y-axis) across different rat chromosomes. [file 13072_2020_376_MOESM5_ESM.png]
